# Using Geovisualization Tools to Examine Attitudes towards Alcohol Exposure in Urban Environments: A Pilot Study in Madrid, Spain

**DOI:** 10.3390/ijerph19159192

**Published:** 2022-07-27

**Authors:** Andrea Pastor, Xisca Sureda, Roberto Valiente, Hannah Badland, Macarena García-Dorado, Francisco Escobar

**Affiliations:** 1 Public Health and Epidemiology Research Group, School of Medicine, University of Alcalá, Campus Universitario-Ctra. de Madrid-Barcelona, Km. 33,600, 28871 Madrid, Spain; andrea.pastor@uah.es; 2 Department of Epidemiology & Biostatistics, Graduate School of Public Health & Health Policy, City University of New York, 205 E 42nd St., New York, NY 10017, USA; 3 Tobacco Control Research Group, Institut d’Investigació Biomèdica de Bellvitge-IDIBELL, l’Hospitalet de Llobregat Avinguda de la Granvia de l’Hospitalet, 199, L’Hospitalet de Llobregat, 08908 Barcelona, Spain; 4 Centro de Investigación Biomédica en Red de Enfermedades Respiratorias (CIBERES), 28029 Madrid, Spain; 5 Centre for Research on Environment, Society and Health (CRESH), School of GeoSciences, University of Edinburgh, Edinburgh EH8 9XP, UK; roberto.valiente@ed.ac.uk; 6 SPECTRUM Consortium, UK; 7 Health, Place and Society Group, Centre for Urban Research, RMIT University, Melbourne, VIC 3000, Australia; hannah.badland@rmit.edu.au; 8 Department of Geology, Geography and Environmental Sciences, University of Alcalá, 28801 Madrid, Spain; garciadoradomacarena@gmail.com (M.G.-D.); francisco.escobar@uah.es (F.E.)

**Keywords:** alcohol environment, GIS, alcohol normalization, scenarios, urban planning, simulation, public health

## Abstract

Pervasiveness of alcohol products and their promotion in the urban landscape may normalize alcohol consumption. This study aims to utilize geovisualization-based methods to assess attitudes towards different levels of alcohol exposure in the urban environment. We selected a typical downtown location, Lavapiés Square in Madrid, Spain, to conduct our study. First, we designed and created realistic 3D models simulating three different urban scenes with varying degrees of exposure to alcohol in the environment. Second, we used a survey on 159 adults to explore the level of acceptance of, attitudes towards, and perceptions of alcohol exposure in each scene. Participants reported a higher level of comfort in the scene with null alcohol exposure compared with the other scenes (*p* < 0.001). Acceptance towards alcohol exposure decreased as the level of alcohol elements increased in the scenes (*p* < 0.01). Acceptance also decreased when children were present in the scenes (*p* < 0.01). This study demonstrated that geovisualization tools provide a useful and well-suited approach to analyze perceptions of the alcohol environment. The use of geovisualization can help understand attitudes and perceptions towards the alcohol environment and may offer a way to simulate different scenarios prior to development or retrofitting.

## 1. Introduction

Worldwide, three million deaths per year result from harmful use of alcohol, representing 5.3% of all deaths [1,2]. The World Health Organization has identified the acceptance of alcohol consumption as a major public health challenge that must be addressed in the coming years [1].

Since the 1990s there has been increasing interest in how built environment features shape individual health behaviors [3,4]. Researchers have identified three built environment dimensions that can influence alcohol behaviors among those who are exposed, namely to alcohol promotion, alcohol availability, and signs of alcohol consumption [5,6,7,8]. Alcohol promotion in the built environment includes ‘front-of-shop’ presence at alcohol outlets and advertising in the streetscape. Higher alcohol availability (including a higher number of alcohol outlets in the environment) may trigger an increase in market competition, resulting in price reductions in alcohol products and therefore enhancing affordability and potentially consumption [6,9,10,11,12,13]. Signs of alcohol consumption include litter (e.g., discarded bottles) and people drinking alcohol outside on street terraces [8,14,15]. Evidence suggests that the presence of these alcohol elements in the urban environment may modify alcohol-related beliefs and social norms by increasing acceptance and normalization of alcohol consumption [16,17,18,19,20].

There is a growing consensus among researchers and policy makers regarding the need to develop effective policy interventions that minimize excessive alcohol consumption [1,21,22]. Interventions regulating the availability, advertising, and pricing of alcohol have been described as some of the most cost-effective strategies to decrease alcohol acceptance and consumption [23]. However, it is important to ensure that the population supports these kinds of interventions in order to maximize their success [24,25].

Interventions targeting alcohol policies have been examined in previous studies. They typically use methodologies based on secondary databases, social systematic observation, and surveys [26,27,28]. However, assessing the acceptability of potential interventions before implementation could ensure that they are more fit-for-purpose, thereby maximizing their effectiveness once applied. The use of geovisualization techniques associated with Geographic Information Systems (GIS) allows us to design and build realistic scenes (or images) to represent the changes in the urban landscape that a given policy intervention may introduce (e.g., banning alcohol advertising and promotion in public outdoor spaces).

Geovisualization refers to a set of tools and techniques that allow the analysis of geospatial data and the creation of scenes to represent spaces and landscapes by using interactive visualization. Geovisualization includes data on different environmental features, such as terrain, buildings, living beings, animation, static and moving objects, natural or human-made, and lighting effects [29]. Previous public health studies have applied geographic visualization techniques to explore exposure to unhealthy environments. For example, Valiente and colleagues used GIS and cartographic outputs to describe and evaluate the visibility of tobacco elements (e.g., smokers and cigarette butt littering) in the environment [30,31,32]. However, to our knowledge, geovisualization tools have not been applied to study the alcohol environment.

Thus, the present manuscript represents a pilot study, which aims to examine the appropriateness application of geovisualization-based methods to assess acceptance of and attitudes towards different levels of alcohol exposure in urban environments. Particularly, this study was developed in Lavapiés Square, a centrally located square in the city of Madrid, Spain.

## 2. Materials and Methods

### 2.1. Study Settings and Design

This pilot study was conducted in the city of Madrid, Spain, in 2017. The use of 3D-modeling required the selection of a specific area of Madrid. We chose Lavapiés Square as the case study for this research. This area is located in “Embajadores” neighborhood in the “Centro” District. Lavapiés Square is representative of any centrally located square in a typical European city: a crowded and busy space with public transportation stops (buses and subway) and a range of retail and leisure venues. We selected this space because it is a well-known location in Madrid, commonly frequented by residents and tourists. Therefore, it was likely that all participants would be familiar with the study area.

**Figure 1:**
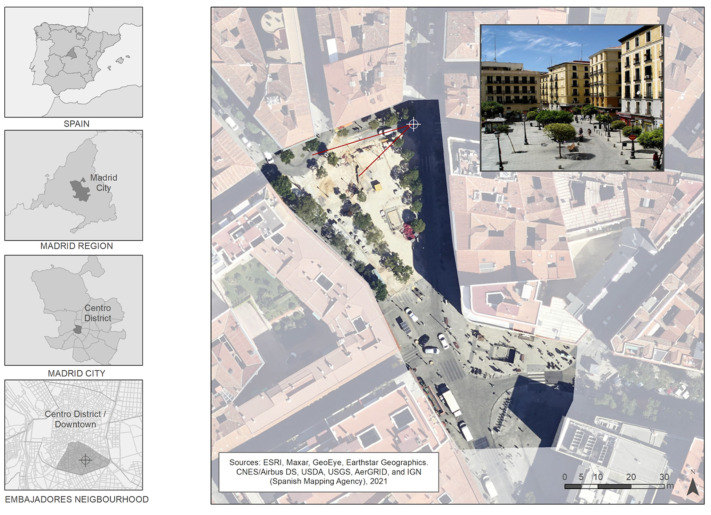
Study area—Lavapiés Square (Embajadores neighborhood, Centro district, city of Madrid). Data source: Spanish Mapping Agency, 2021.

Figure 1 shows the location of this study setting within the city of Madrid.

Lavapiés Square is designed as an open space of irregular layout. We identified five bars and restaurants with terraces and six off-premise alcohol outlets in Lavapiés Square. The data were gathered from the Municipal Census of commercial and business establishments and validated on-field in 2017. Further validations conducted in 2021 using Google Street View determined that these exposures remained the same at both time points. We also observed alcohol promotion associated with the alcohol outlets (e.g., Billboards) and visible signs of alcohol consumption in the street, especially alcohol-related litter.

The study was organized in two steps. First, we designed and implemented three 3D-scenes to represent different degrees of presence, visibility of alcohol promotion, and signs of alcohol consumption in Lavapiés Square. Second, we developed and conducted a survey to assess the level of acceptance, attitudes, and perceptions of participants using these three pre-defined scenes.

### 2.2. Generation of the 3D Scenes

Although there are different and often contradictory definitions of ‘scenarios’ [33], in this study we used the definition provided by the International Permanent Committee for Climate Change 2000 Report, summarized by Escobar et al., (2017, p. 82). Accordingly, a scenario was defined as the exploration of multiple plausible situations with the purpose of assisting in the understanding of possible future developments of complex systems. The concept of the scene referred to the materialization of a given scenario into a pictorial representation by means of realistic 3D techniques [33]. Thus, in this study, we designed three 3D-scenes, each reflecting a different scenario of alcohol exposure (e.g., null alcohol exposure, medium alcohol exposure, and high alcohol exposure).

We created a geographical 3D model of Lavapiés Square using geovisualization to generate the three alcohol-related scenes. According to Cauvin et al. (2010), the 3D models were composed of fixed, permanent elements (e.g., topography, vegetation, built-up structures) and mobile elements (e.g., human beings, motor vehicles). These elements were complemented with non-tangible elements, such as lighting and atmospheric conditions. Permanent and mobile elements needed to generate the scenes were retrieved from the following data sources and managed individually:

- Streets and public pathways were downloaded from the Open Street Map (OSM) database (https://www.openstreetmap.org/, accessed on 26 July 2022) in 2017.
- Buildings (including their footprints and heights) were downloaded from the Spanish Land Registry (Cadastre, https://www.sedecatastro.gob.es, accessed on 26 July 2022) in 2017.
- Information on the location of the entrances to the on- and off-premise alcohol outlets (expressed by Universal Transversal Mercator (UTM) coordinates and address) bordering Lavapiés Square were obtained in 2017 from the Municipal Census of commercial and business establishments, available at the Open Database of the Madrid City Council (https://datos.madrid.es/portal/site/egob, accessed on 26 July 2022). The dataset was validated by field observation in 2017. A second validation in 2021 using Google Street View identified that the number of on- and off-premises alcohol outlets had not changed in the intervening time.
- Vegetation information (e.g., trees located within the study area) and urban furniture elements (e.g., benches, lampposts, refuse containers, bus stops, kiosks) were identified and georeferenced from satellite and street-level images using the Google Earth Platform (https://www.google.com/intl/es/earth/, accessed on 26 July 2022) in 2017.
- The location of other mobile elements (e.g., pedestrians, cars) throughout the 3D model and the scenes was arbitrarily assigned.

**Figure 2:**
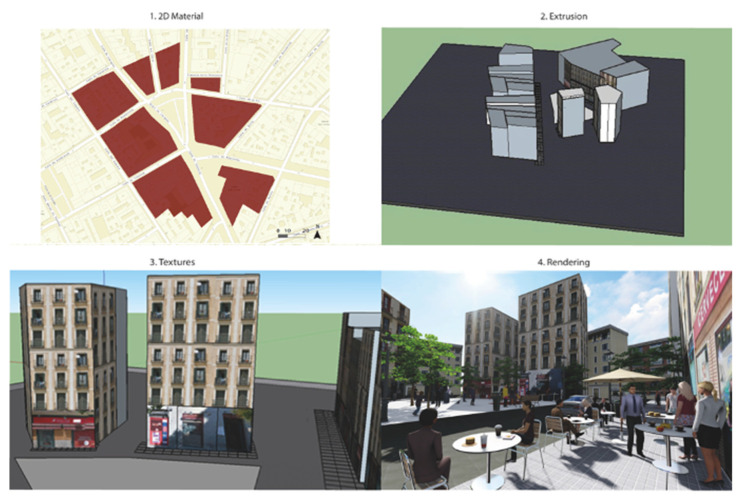
3D modeling procedures (2D Material, Extrusion, Textures, and Rendering).

The process adopted for the creation of the scenes was organized in four steps, summarized in Figure 2.

(1) All data described earlier (e.g., streets, buildings, vegetation, urban furniture, and other mobile elements) were integrated into a geodatabase and georeferenced the information on a 2D map.
(2) A Digital Elevation Model (DEM) was created to convert the 2D map into a 3D model. A DEM is a 3D model that includes data elevation data for each element included in the map. In our study, we did not include topographic information to build the 3D model, since the Lavapiés Square was small (around 0.41 ha). Moreover, we regarded the ground surface as a flat terrain. Therefore, the DEM was generated by extruding the building polygons using the elevation (height) attribute assigned to each feature (provided by the Cadastre).

Both the first and second steps were conducted using ArcGIS v10.4 software (ESRI, Redlands, CA, USA, https://www.arcgis.com/index.html, accessed on 26 July 2022).

(3) Each building extruded polygon was exported to SketchUp v.20.0 software (Trimble, CA, USA, https://www.sketchup.com/, accessed on 26 July 2022) in COLLADA format to design and model the facades of the buildings. This process is known as “building texturing”. We used original photographs taken on-field to complete the building texturing for each feature.
(4) Once the texturing process was completed, we added the textured buildings into our 3D model via GIS software and exported it into Lumion v.10 software (Warmond, The Netherlands, https://lumion.com/, accessed on 26 July 2022) to depict the vegetation, urban furniture, and the mobile elements. We used different models imported from Lumion libraries (https://lumion.com/lumion-content.html, accessed on 26 July 2022) and SketchUp (https://3dwarehouse.sketchup.com/, accessed on 26 July 2022) for each feature (e.g., trees, pedestrians, benches). We also used Lumion software to edit illumination properties and render our model to provide a more realistic appearance.

A representative view angle model was selected (see Figure 1) that was exported in image format (.tiff). Adobe Photoshop (Adobe, Mountain View, CA, USA, (https://www.adobe.com, accessed on 26 July 2022) was used to add different alcohol-related elements to the scenes (e.g., bottles, glasses, banners, people consuming alcoholic beverages).

The scenes were designed according to a strategy based on a progressive incorporation of alcohol-related elements developed by a group of experts on alcohol environments [34]. Scene 1 did not include any exposure to alcohol elements except for the presence of alcohol outlets (null alcohol exposure scenario). Scene 2 included some alcohol-related elements (medium-exposure scenario). Scene 3 was characterized by the highest amount of alcohol products, alcohol promotion, and signs of consumption in alcohol outlets, both on and off premises and in other public spaces (high-exposure scenario).

### 2.3. Resemblance, Degree of Comfort, and Acceptability towards the Scenes

#### 2.3.1. Survey Design

The survey (refer to Supplementary Materials for the full survey) measured the degree of re-semblance to Lavapiés Square (low, medium, or high) and the participants’ level of comfort with each alcohol exposure scene (questions A1 to A2 and A3 to A4 in Supplementary Materials, respectively). Level of comfort was defined as being how safe, secure, and welcome the participants would feel at each scene. We also assessed whether participants would visit the scene with and without children (questions A5 in Supplementary Materials). The items were scored from 1 (least comfortable/least accepted) to 10 (most comfortable/accepted). Participants’ sociodemographic information was collected in the final part of the survey. Participants reported their age, sex, place of residence, number of children living in their household, and educational attainment. The survey was prepared and administered using Google Forms (https://www.google.es/intl/es/forms/about/, accessed on 26 July 2022) and the link was distributed by email between June and July 2017.

#### 2.3.2. Sampling of Participants

We used convenience sampling methods and invited participation by staff at two departments of the University of Alcalá (Spain), namely Epidemiology and Public Health, Geology, Geography, and Environmental Sciences. From these responses, we contacted 47 participants and invited them to complete the survey and share the survey invitation with their relatives and colleagues, initiating a non-probabilistic snowball sampling. We selected the snowball sampling technique since there was no obvious population of interest. In addition, we considered snowball sampling as a helpful method to capture valuable preliminary information about a population that was, in principle, unknown [35].

A total of 159 (84 women, 75 men) participants completed the survey. Overall, 47.6% of the participants were aged between 18 and 30 years and 74.2% reported having a university education. All participants resided in the region of Madrid, ensuring familiarity with Lavapiés Square.

#### 2.3.3. Statistical Analysis

A descriptive analysis was conducted examining the participants’ responses to alcohol acceptance and attitudes to the different scenes. We computed proportions for qualitative items and medians for quantitative items. Accumulated frequency graph bars were generated to examine the resemblance of each scene to the alcohol exposure found in Lavapiés Square. We also produced box plots summarizing the degree of comfort and acceptability with each scene.

None of the variables followed parametrical distribution. Therefore, chi square tests were used to analyze the differences of resemblance with reality between the scenes. The remaining variables were analyzed by Kruskal–Wallis tests. All the statistical tests have a significance level of 95%. The software used for the analysis was STATA v. 12.0 (Data Analysis and Statistical Software).

## 3. Results

### 3.1. Final Scenarios of Alcohol Exposure

**Figure 3:**
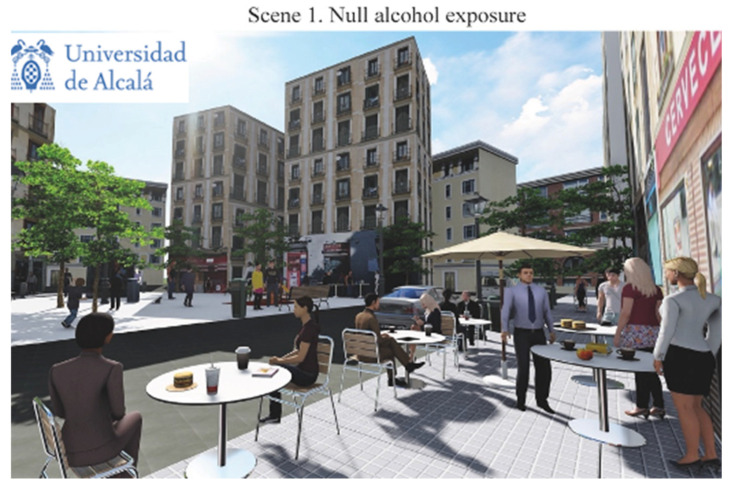
Null alcohol exposure scenario.

**Figure 4:**
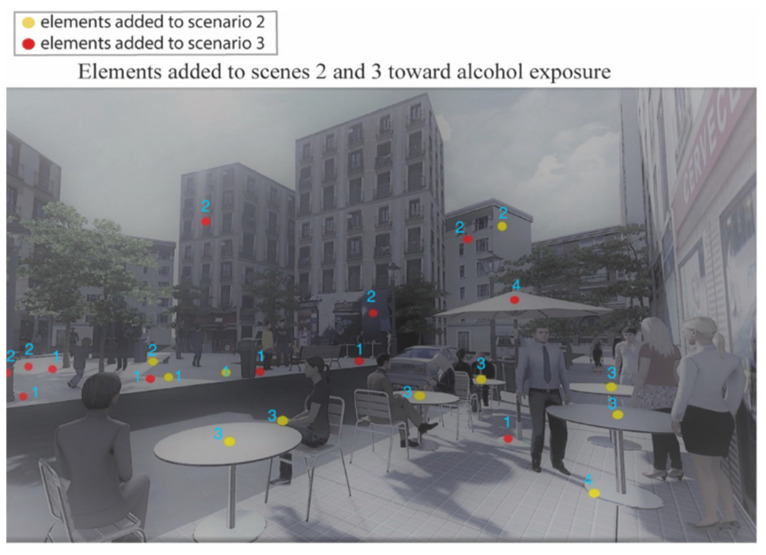
Points where we added alcohol elements (yellow points: medium-exposure scenario; red points: high-exposure scenario). Alcohol elements included: (1) litter related with alcohol; (2) displays with alcohol promotion; (3) alcoholic beverages (or people consuming alcohol); and (4) alcohol promotion associated to alcohol outlets.

**Figure 5:**
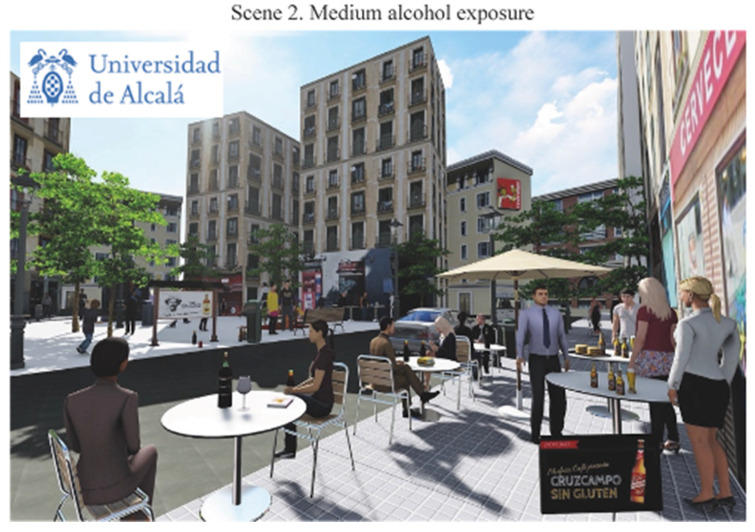
Medium alcohol exposure scenario.

**Figure 6:**
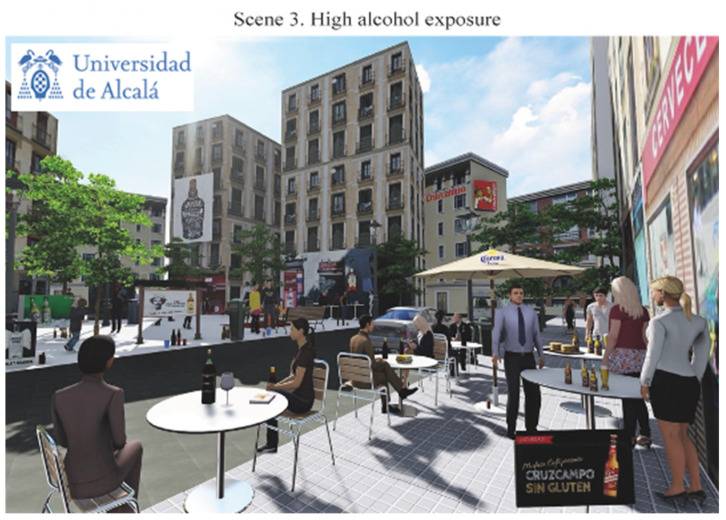
High alcohol exposure scenario.

The final scenes representing different alcohol exposure scenarios in the Lavapiés Square obtained are listed below in the following order: Figure 3 shows null alcohol exposure scenario (Scene 1); Figure 4 shows the points in where we modified the levels of exposure; Figure 5 shows the medium alcohol exposure scenario (Scene 2): and Figure 6 shows high level of alcohol exposure scenario (Scene 3).

Scene 1 (Figure 3) represents the null alcohol exposure scenario. In this scene, no elements of alcohol promotion nor signs of consumption were included. Only on-and off-premise alcohol outlets that were presently available in Lavapiés Square were maintained. Moreover, we included people walking on the street but none of them were drinking alcohol. The people presented in the scene were gender-balanced (9 women and 10 men), and most of them were representing young and middle-aged professionals (89.9%), identifiable by wearing well-dressed clothing. These elements were used to try to reflect that Lavapiés Square is a popular destination for people to drink and socialize after work. Moreover, we depicted a group of children playing at the back of the Square. As noted in the Section 1, the representation of children played a key role in all the scenes, as they are vulnerable group, and we were interested in assessing perceptions of alcohol exposure when children were present.

The differences amongst the scenes are highlighted in the Figure 4.

Scene 2 (Figure 5) represents the medium alcohol exposure scenario. In contrast to scene 1, scene 2 included some elements related to alcohol consumption. This scene included three elements of alcohol promotion. All the promotion images used in the scene represented local well-known alcohol brands. Scene 2 also included two alcohol litter elements and six elements representing people consuming alcohol in on-premise terraces (11 elements in total, see yellow dots in Figure 4). We represented all the people consuming alcohol in this scene as young and middle-aged professionals. All of them were drinking beer or wine beverages, as these types of alcohol beverages are most commonly consumed in Spain [36].

Finally, scene 3 (Figure 6) represented the highest alcohol exposure scenario. To create this scene, 12 additional alcohol elements were added to scene 2 (see red dots in Figure 4). In brief, we increased the exposure to alcohol promotion by adding five displays. Three billboards were places on the buildings (two related with beer brands and one with a spirit beverage) and the rest were located at one side of the square (one with a beer brand and the other with a spirit beverage) where the children were playing. In addition, we included one beer brand on a terrace umbrella. The ‘branded umbrella’ attempted to represent a very common marketing practice in Spain, where alcohol wholesalers and providers lend branded furniture to on-premise alcohol outlets so they are able to furnish their venues and terraces [37,38]. Similarly, the exposure to alcohol litter was increased by inserting five elements within the Square. No additional people were represented consuming alcohol in scene 3.

### 3.2. Resemblance and Degree of Comfort and Acceptability towards the Scenes

#### 3.2.1. Resemblance of Each Scene with the Alcohol Exposure That Can Be Found in Lavapiés Square

The three scenes were included in the survey. All the elements depicted in the scenes were correctly understood and interpreted by the participants, and all participants completed the questions.

**Figure 7:**
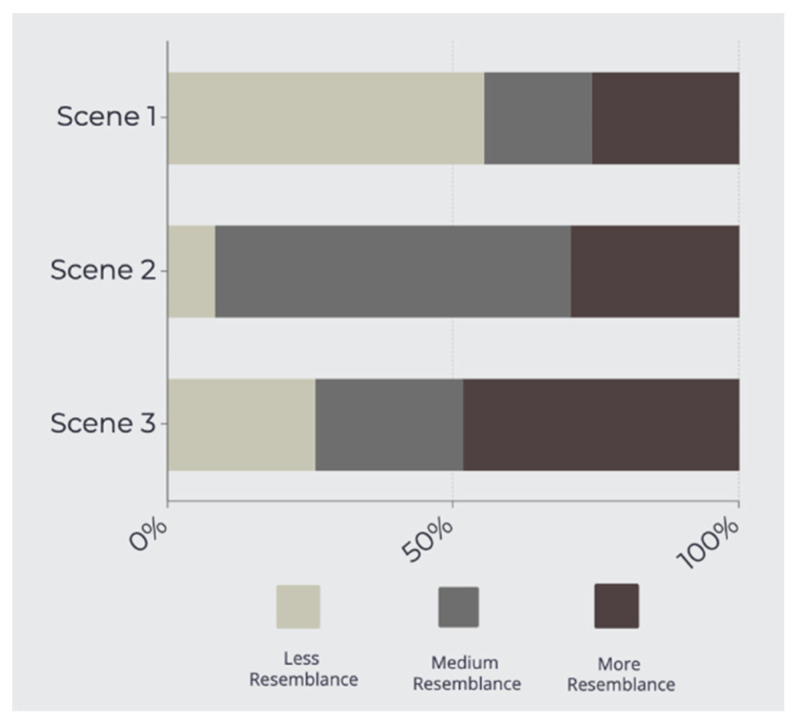
Resemblance of each scene with the alcohol exposure that can be found in Lavapiés Square in real-life conditions (Scene 1: null alcohol exposure; Scene 2: medium alcohol exposure; Scene 3: high alcohol exposure).

Figure 7 shows the results obtained for the perceived resemblance of each scene with the alcohol exposure in Lavapiés Square.

Overall, the scenario with null alcohol exposure (scene 1) had the lowest percent of perceived resemblance (25.79%) with Lavapiés Square, followed by the scenario with medium alcohol exposure (scene 2). The majority of participants identified that the scenario with the highest alcohol exposure (scene 3) was most like the alcohol exposure found in Lavapiés Square (48.4%).

#### 3.2.2. Degree of Comfort Participants Would Feel at Each Scene

**Figure 8:**
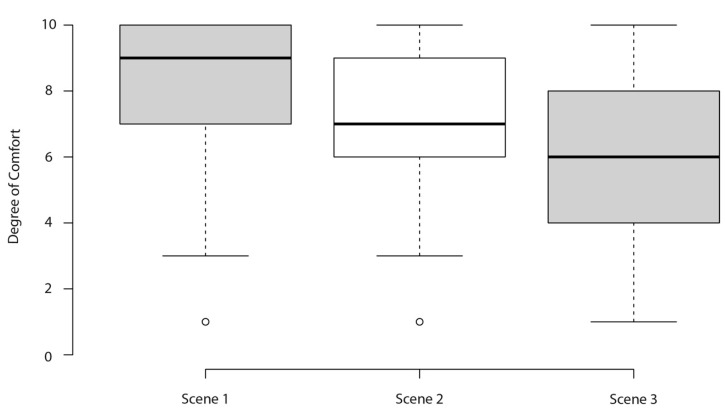
Reports of participants’ level of comfort in each of the scenarios. Scene 1: null alcohol exposure; Scene 2: medium alcohol exposure; Scene 3: high alcohol exposure. *p*-value assessed with Kruskal–Wallis.

The level of comfort perceived by the participants for each scene was shown in Figure 8. We found statistically significant median score differences between the scenes (*p* < 0.001). On a scale of 1 to 10 (10 maximum comfort), the scenario with null alcohol exposure (scene 1) had a median score of 9 (IQR: 7−10), indicating the scene with the highest level of perceived comfort. The scenario with medium alcohol exposure (scene 2) had a median score of 7 (IQR: 6−9), and the scenario with high alcohol exposure (scene 3) had the lowest median score of perceived comfort (median = 6 (IQR: 4−8).

#### 3.2.3. Acceptability for Visiting Each Scene (with/without Children)

**Figure 9:**
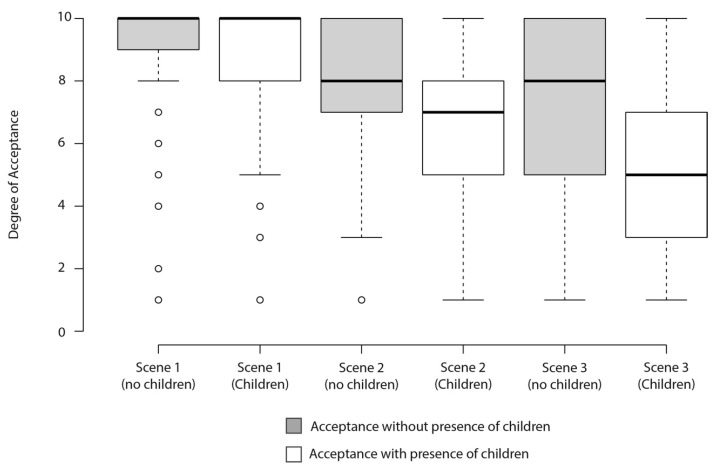
Median scores for level of acceptability in each scenario, with and without children. Scene 1: null alcohol exposure; Scene 2: medium alcohol exposure; Scene 3: high alcohol exposure *p*-value assessed with Kruskal–Wallis.

The results related to acceptability of visiting each of the scenarios (with and/without children) were shown in Figure 9. Participants showed significantly higher levels of acceptance for visiting the null alcohol exposure scenario without children, compared with the other two potential scenarios (median values of 10 (IQR: 7−10, null alcohol exposure), 8 (IQR: 7−10, medium alcohol exposure), and 8 (IQR: 5−10, high alcohol exposure)). Similarly, the degree of acceptance of visiting the null exposure scenario with children was the highest and showed a sharp decrease as the higher alcohol exposure scenarios were considered (median scores of 10 (IQR: 8−10), 7 (IQR: 5−8), and 5 (IQR: 3−7) for the null, medium, and high alcohol exposure scenarios, respectively).

## 4. Discussion

This pilot study explored the potential use of geovisualization-based approaches to assess attitudes towards different levels of alcohol exposure in an urban setting. Geovisualization tools have been widely used in landscape, spatial planning, and environmental impact research [39,40,41,42,43,44]. For example, building and disseminating realistic virtual scenes with the purpose to communicate specific urban planning information (e.g., new housing developments in a city, changes in the street network design, etc.) and environmental issues (e.g., concentration of air pollutants derived from vehicle emissions, estimation of areas with high risk of flooding, etc.) [41,42,43,44,45]. The virtual scenes can be yielded in 2D and 3D images or videos, which can provide an interactive and immersive experience to the users (e.g., both policy-makers and citizens) to help understand, imagine, and discuss different past or future scenarios [42,43]. Thus, geovisualization tools offer the possibility to create user-centered experiences [46], and for this reason, they have been employed to gather information on perceptions of the urban physical and social environment [44] and to facilitate the decision making process [40,47]. Similarly, given their interactive, autonomy, and immersive characteristics, the potential of geovisualization for the creation of participatory solutions in citizen science approaches has been identified [46]. It is also important to note that the latest advances in computation, virtual reality, and web mapping have facilitated the creation and implementation of geovisualization tools to extend GIS capabilities and to create geographic Immersive Virtual Environments (also referred as GeoIVE in the literature) [43,44,48].

Overall, the use of GIS has been progressively applied to the public health field. For example, estimating the availability of, accessibility to, and exposure to unhealthy commodities, such as alcohol, in the environment [49,50,51], or assisting in the creation of cartographic dashboards (e.g., web-mapping platforms) to display useful data for surveillance and/or policy guidance [52,53]. However, the use of geovisualization methods have typically not been integrated into public health, and its potential benefits derived from its implementation remain unknown [31,32].

In the present study, we used geovisualization to create scenarios with different levels of neighborhood alcohol exposure. These scenarios could readily be adapted to test implementation of potential policies; in this case, policies to reduce alcohol exposure in the environment. However, because of its flexibility, geovisualization could also be applied to other exposures of policy interest (e.g., tobacco, fast food). Moreover, geovisualization enabled us to create fit-for purpose scenes and test elements that were of interest (e.g., alcohol promotion billboards, people consuming alcohol, alcohol-related litter), while minimizing the presence of less-relevant aspects (e.g., noise) [47]. For example, we minimized car-traffic elements in our sense as they constitute mobile/dynamic elements that might conflate perceptions of alcohol-related elements in our study area [29].

Differences across the three scenes were understood by the participants, and geovisualization proved to be helpful for visualizing the topics measured by the survey. Participants reported the scenario with the highest alcohol exposure (scene 3) as most resembling the present level of alcohol exposure in Lavapiés Square. These results might be interpreted as a reflection of the high level of exposure to alcohol in our case study site, which is indeed highly normalized by our study sample of participants. Participants also identified the scenario with null alcohol exposure (scene 1) as being most acceptable to them. However, the scores obtained for the acceptability of visiting the medium and high alcohol exposure scenarios remained high (8/10 and 8/10, respectively). According to Social Cognitive Theory, constant exposure to a wide variety of alcohol elements in the environment potentially increases the normalization and acceptance of alcohol products and its consumption [54,55]. For example, high levels of alcohol exposure can lead towards promoting more positive assumptions and beliefs regarding alcohol consumption. Moreover, the ubiquity of alcohol promotion can generate an automatic cognitive process, whereby high levels of exposure mean people perceive these elements to be part of their daily lives and, as a consequence, underestimate overall alcohol exposure [56,57,58,59]. Indeed, previous studies have found positive associations between the presence of alcohol in the streetscape (e.g., alcohol advertising, alcohol availability, and different signs of alcohol consumption) and its normalization [16,18], which is in line with our findings.

The results also suggest that a setting with higher levels of alcohol exposure becomes less acceptable when children are present. The reluctance to expose children to alcohol potentially provides an opportunity to implement policies that regulate alcohol exposure in urban settings, which may help prevent young people from consuming alcohol [60,61].

Our findings support that geovisualization techniques can generate realistic alcohol exposure scenes and provide a valuable tool to study the population’s awareness and perception towards alcohol. Moreover, the use of this method provides useful avenues for identifying policy levers to reduce alcohol exposure. Effective examples of alcohol policies include capping the availability of alcohol outlets, limiting the presence of alcohol promotion in the street, or promoting educational campaigns to improve population sensitivity on alcohol-related issues [17,28,62,63]. Our findings align to these policies as the majority of participants identified the scene 1 (null alcohol exposure) as being the most desirable, especially in the presence of children. Therefore, the implementation of policies that regulate alcohol exposure around youth-serving facilities (e.g., schools, playgrounds) may be worthwhile to pursue [18,49]. One example of this type of policy is in Edmonton, Canada, where the location of alcohol outlets are restricted within 100 m from schools [64].

Although this is a pilot study with promising results, some limitations exist. First, the use of fixed images to capture the scenes in the survey meant that other sensory factors, such as hearing and smell, were ignored. Future studies could take advantage of the latest advances in virtual reality and Internet technologies to explore the use of video instead of presenting static images/scenes. Second, we used a snowball technique to select our participants. Recruiting through a university and attempts to circulate the survey through participant networks likely introduced bias to our sample. However, as a pilot study, we are confident that we demonstrated face validity of our geovisualization-based approach.

Despite these limitations, this study shows innovative implementation of geovisualization methods to explore perceptions and attitudes towards alcohol exposure in the urban European environment. To our knowledge, this is the first study using a realistic geovisualization-based approach to assess the acceptance of different levels of alcohol exposure in an urban setting. This study demonstrated that the use of geovisualization tools constituted a useful and well-suited approach for investigating perceptions of the alcohol environment in an urban setting, allowing the creation and testing of hypothetical scenarios before developing any potential intervention.

## 5. Conclusions

This study shows the ubiquity of alcohol elements in the urban environments. Our results describe the benefits and advantages of using geovisualization to understand the perceptions of and potential for assessing a local alcohol environment. In our sample, the acceptability of alcohol exposure was high; however, alcohol-free environments were more acceptable, especially in the presence of children. As discussed in this study, this methodology represents an opportunity for policy makers and practitioners to design, develop, and test potential interventions and policies in an effort to reduce alcohol acceptance and its consumption.

Beyond the alcohol environment exposure, geovisualization tools offer a novel approach to better identify and assess the exposures to other physical or social features in urban settings associated with other unhealthy commodities and habits (e.g., tobacco, fast food, physical inactivity, or gambling). Similarly, the use of geovisualization in combination with GIS and other emerging virtual reality techniques, such as the GeoIVE, may allow a higher degree of flexibility for researchers to create a wide range of scenes characterizing and representing daily social situations and activities related to the development of certain unhealthy habits (alcohol drinking, smoking, unhealthy eating) rooted in certain places with particular characteristics. Specifically, this might represent a key methodological step to understand how exposure to certain urban features relates to unhealthy habits. Furthermore, as shown in this piece of work, the simulation of these scenes might assist in the designing of appropriate interventions in the physical environment to improve people health, as well as test the degree of support of these interventions among residents, which is crucial to ensure their adequateness and effectiveness.

## Data Availability

Not applicable.

## Supplementary Materials

The following supporting information can be downloaded at: https://www.mdpi.com/article/10.3390/ijerph19159192/s1. This document includes the survey used in this study. This survey measured the degree of resemblance to Lavapiés Square (low, medium, or high) and the participants’ level of comfort with each alcohol exposure scene.
