# Supplementary material for: Using Geovisualization Tools to Examine Attitudes towards Alcohol Exposure in Urban Environments: A Pilot Study in Madrid, Spain
Source: Int J Environ Res Public Health. 2022 Jul 27;19(15):9192. doi: 10.3390/ijerph19159192 (PMC9368102; doi:10.3390/ijerph19159192)
Supplement: Supplementary file 1 [file ijerph-19-09192-s001.zip › ijerph-1747903-supplementary.pdf]

Supplementary Material- survey[i]

A. Please look at the scenes below and answer the following questions:

A1. Rate on a scale of 1 (least) to 10 (most) the presence of alcohol-related elements in each of the scenes.

**Scene 1**	1	2	3	4	5	6	7	8	9	10
**Scene 2**	1	2	3	4	5	6	7	8	9	10
**Scene 3**	1	2	3	4	5	6	7	8	9	10

A2. Rate on a scale of 1 (least) to 3 (most) the resemblance between the scenes and the reality of Madrid, specifically Lavapiés Square, in terms of the presence of alcohol-related elements.

**Scene 1**	1	2	3
**Scene 2**	1	2	3
**Scene 3**	1	2	3

A3. Rate on a scale of 1 (least comfortable) to 10 (most comfortable) how you would feel in each of the scenes.

**Scene 1**	1	2	3	4	5	6	7	8	9	10
**Scene 2**	1	2	3	4	5	6	7	8	9	10
**Scene 3**	1	2	3	4	5	6	7	8	9	10

A4. Would you accept being in these scenes? (1=least acceptance, 10=greatest acceptance).

**Scene 1**	1	2	3	4	5	6	7	8	9	10
**Scene 2**	1	2	3	4	5	6	7	8	9	10
**Scene 3**	1	2	3	4	5	6	7	8	9	10

A5. In the presence of children, would you accept being in these scenes? (1=least acceptance, 10=greatest acceptance).

**Scene 1**	1	2	3	4	5	6	7	8	9	10
**Scene 2**	1	2	3	4	5	6	7	8	9	10
**Scene 3**	1	2	3	4	5	6	7	8	9	10

B. To conclude, please fill out this section on sociodemographic information.

B1		Your age
	1	Under 18
	2	18-30
	3	31-60
	4	Over 60

B2	Your sex
	Man
	Woman

B3		Do you live in Madrid?
	1	Yes ► Go to question C4
	2	No ► Go to question C5

B4		In what neighborhood of Madrid do you live?

B5		If you do not live in Madrid, in which city do you live?

B6		Number of children you have
	1	None
	2	1
	3	2
	4	3 or more

B7		What is your maximum level of completed studies? (Mark only one option)
	1	You know how to read and to write but you attended school for less than 5 years
	2	Unofficial Primary Education
	3	Official Certificate of Primary Education
	4	Secondary Education
	5	Professional Secondary Education
	6	Professional basic tertiary education
	7	University Bachelors Degree
	8	University Honors Degree
	9	University Masters degree
	10	PhD
	11	Other (please specify)

[i] The original questionnaire was written and sent out in Spanish. This is a translated version for its publication in International Journal of Environmental Research and Public Health
